# Assessing immunological and virological responses in the liver: Implications for the cure of chronic hepatitis B virus infection

**DOI:** 10.1016/j.jhepr.2022.100480

**Published:** 2022-04-02

**Authors:** Tobias Boettler, Upkar S. Gill, Lena Allweiss, Teresa Pollicino, John E. Tavis, Fabien Zoulim

**Affiliations:** 1 Department of Medicine II (Gastroenterology, Hepatology, Endocrinology and Infectious Diseases), Freiburg University Medical Center, Faculty of Medicine, University of Freiburg, Freiburg, Germany; 2 Blizard Institute, Centre for Immunobiology, Barts and the London School of Medicine and Dentistry, Queen Mary University of London, London, UK; 3 I. Medical Clinic and Polyclinic, University Medical Center Hamburg-Eppendorf, Hamburg, Germany; 4 German Center for Infection Research (DZIF), Hamburg-Lübeck-Borstel-Riems sites, Germany; 5 Department of Human Pathology, University Hospital "G. Martino" of Messina, Messina, Italy; 6 Department of Molecular Microbiology and Immunology and Institute for Drug and Biotherapeutic Innovation, Saint Louis University, Saint Louis MO USA; 7 INSERM Unit 1052 – Cancer Research Center of Lyon, Department of Hepatology Hospices Civils de Lyon, Lyon University, France

**Keywords:** liver biopsy, fine needle aspirates, biomarker, viral hepatitis, HBsAg, cccDNA, viral integration, T cells, B cells, cccDNA, covalently closed circular DNA, CHB, chronic hepatitis B, FNA, fine needle aspiration, HBcrAg, HBV core-related antigen, pgRNA, pregenomic RNA

## Abstract

Cure from chronic HBV infection is rare with current therapies. Basic research has helped to fundamentally improve our knowledge of the viral life cycle and virus-host interactions, and provided the basis for several novel drug classes that are currently being developed or are being tested in clinical trials. While these novel compounds targeting the viral life cycle or antiviral immune responses hold great promise, we are still lacking a comprehensive understanding of the immunological and virological processes that occur at the site of infection, the liver. At the International Liver Congress 2021 (ILC 2021), a research think tank on chronic HBV infection focused on mechanisms within the liver that facilitate persistent infection and looked at the research questions that need to be addressed to fill knowledge gaps and identify novel therapeutic strategies. Herein, we summarise the discussion by the think tank and identify the key basic research questions that must be addressed in order to develop more effective strategies for the functional cure of HBV infection.

**Key points**

• Current therapies for chronic HBV infection are not curative.
• Several novel drug classes are currently being developed or tested in clinical trials.
• We are still lacking a comprehensive understanding of the immunological and virological processes that occur in the liver.
• At the International Liver Congress 2021 (ILC 2021), a research think tank organised by EASL and the ICE-HBV (https://ice-hbv.org/) identified several key questions that need to be addressed in HBV research in the immediate future.
• Sampling of the liver, by core biopsies or fine needle aspiration, and standardised readouts are required to advance HBV research.
• Dynamics of cccDNA replenishment and maintenance need to be unravelled in more detail.
• The impact of integrated HBV DNA on novel cure strategies requires further attention.
• The transcriptional activity of cccDNA is still incompletely understood.
• Relevance of intrahepatic and blood-derived immunity on cure strategies needs to be investigated.

## Introduction

Development of curative strategies for chronic HCV infection is a shining example of how basic research can facilitate development of targeted therapies against persistent viral infections. In contrast to HCV, a virus that establishes chronic infection solely by continuous viral replication, HBV also establishes a reservoir of long-lived HBV DNA in infected hepatocytes. This reservoir consists of an episomal viral mini-chromosome called covalently closed circular (ccc)DNA. In addition, some viral DNA also integrates into the host’s genome. The hepatic pool of cccDNA molecules is synthesised by reverse transcription of the viral pregenomic RNA.^1^ Exhaustion of the antiviral immune response, partly due to decade-long exposure to high levels of viral antigens, additionally contributes to viral persistence.^2^^,^^3^

Thus, blocking viral replication alone – a concept that works well in chronic HCV infection – is insufficient to achieve a functional cure (*i.e.* loss of HBsAg and HBV DNA in the serum post treatment withdrawal) in a significant proportion of patients with chronic HBV. Targeting the persistent reservoir in the liver is therefore the most promising approach for novel therapies. However, the strategies by which this goal could be achieved are less clearly defined, partly due to a lack of knowledge about central aspects of the biology of cccDNA and viral integrants, but also of the infected liver immune microenvironment. These open questions range from the generation and transcriptional activity of cccDNA and viral integrants, to the maintenance of infected hepatocytes and their susceptibility to immune-mediated attacks. Addressing these questions will be key to establishing a comprehensive understanding of HBV biology in the liver and outsmarting this sophisticated virus. In this manuscript, we summarise the discussion of the research think tank on chronic HBV infection that took place at the International Liver Congress in 2021, wherein we primarily addressed the importance of investigations of the liver compartment in the development of novel cure strategies.^1^^,^^4^

## Sampling of the liver

### Why does the liver need to be sampled?

**Fig. 1:**
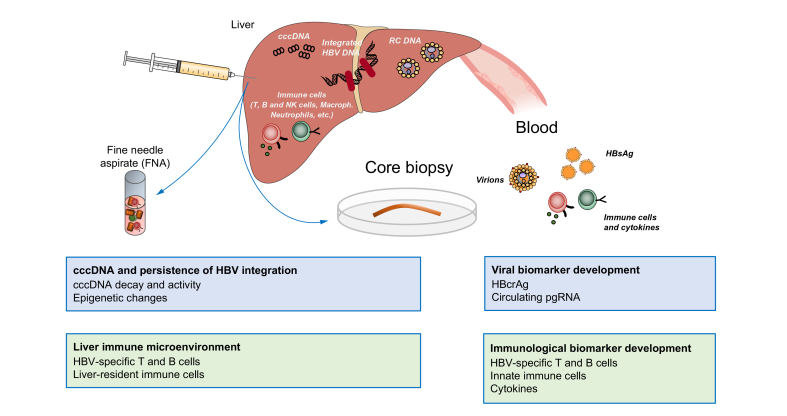
Assessing immunological and virological responses in the liver during chronic HBV infection. Viral persistence is facilitated by several mechanisms that have not been completely unravelled but include the formation of cccDNA, viral integration and the immunological environment. While sampling of the liver, by fine needle aspiration or core biopsies, is of utmost importance to gain a deeper understanding of the precise virological and immunological mechanism that are active at the site of infection, some correlates of transcriptional activity and HBV-specific immunity can also be analysed in the circulation. cccDNA, covalently closed circular DNA; HBcrAg, HBV core-related antigen; NK, natural killer.

Sampling of liver tissue allows for a detailed assessment of liver histology, which may reflect liver damage over time and can aid clinical management of the patient with HBV. Such information cannot be determined with strict accuracy using non-invasive markers similar to those used for chronic hepatitis C and non-alcoholic fatty liver disease,^5^ although they are increasingly used by clinicians in the routine management of chronic hepatitis B.^6^ Validated histological scoring systems in viral hepatitis, Ishak, Knodell and METAVIR, are used to grade disease severity and activity.^7^ Aside from standard diagnostics, additional immunohistochemical tests, such as the distribution of HBsAg and HBcAg may help determine phases of infection and can provide further information on HBV pathogenesis. Assessment of constituents of the HBV reservoir, such as the quantification of cccDNA, requires tissue sampling until new reliable biomarkers are available, though these may be emerging (*e.g.*, HBV core-related antigen [HBcrAg]^8^; circulating pregenomic RNA [pgRNA]^9^). True detection/quantification of cccDNA, assessment of its epigenetic status, and evaluation of integrated HBV DNA will be key in evaluating strategies aimed at the functional cure of chronic hepatitis B (CHB).^10^^,^^11^ Tracking residually infected hepatocytes will be of the utmost importance in ascertaining the effect of new strategies to clear the viral reservoir. Recent advances have demonstrated the presence of immune cell populations in the liver, which are not reflected in blood sampling. These tissue-resident immune cells are responsible for immune surveillance and local tissue immunity, which are key to the immunopathogenesis of HBV.^12^^,^^13^ Their study may provide new insights into the capacity to re-educate the immune microenvironment to control/clear infected hepatocytes. The impact of liver-infiltrating immune cells, both HBV-specific and bystander, also requires further study in the HBV-infected liver, especially during different disease phases and during treatment. Such immune cell analysis will provide further information that may lead to new strategies to probe potential approaches to HBV cure, underscoring the importance of continued sampling of the liver. The integration of virological and immunological data from the liver compartment should provide critical information to evaluate the potential of emerging therapeutic strategies to restore intrahepatic antiviral immune responses and cure viral infection both in treatment-naïve patients and those under virological suppression on nucleos(t)ide analogues (NAs) (Fig. 1).

### Should we perform core biopsies or fine needle aspiration?

At present, direct liver biopsy is required for histological diagnosis and thus remains a standard for diagnosis. With core biopsy, tissue surplus to diagnostic requirements can be utilised to gain information on the viral and immune pathogenesis of hepatitis B. As multiple novel agents are currently being investigated in clinical trials for cure of HBV and organisations such as the International Coalition for Elimination of HBV aim to fast-track safe curative therapies to eliminate HBV globally, longitudinal sampling of the liver compartment will be important to advance the mission of designing curative therapies for HBV. However, core biopsies are invasive and, thus, not usually performed sequentially. A solution for this could be the use of fine needle aspiration (FNA) of the liver. The hepatic FNA technique was first used in 2005 to identify immunological markers in CHB.^14^ Since then, it was shown that FNA can be used to comprehensively sample intrahepatic immunity, covering a broad range of leucocytes, including HBV-specific T cells, tissue-resident immune cells and mononuclear phagocytes. In addition, parenchymal and non-parenchymal cells (Kupffer cells, liver sinusoidal endothelial cells) can also potentially be obtained from FNA and analysed in tandem with other immune cells.^15^ We appreciate that FNA, where only approximately 20,000-50,000 total cells can be obtained per pass, is not the same as liver biopsy and some immune cell markers still require liver tissue sampling. For example, immune cells binding to certain chemokines and integrins that are tightly tethered to the hepatic endothelium may not be sufficiently sampled by FNA. Despite this, accurate sampling of parameters localised in the liver (*e.g.* tissue-resident immune cells, hepatocytes and cccDNA [Testoni/Gill *et al.*, unpublished data]) indicate they could be a good surrogate. Although FNA does not yet provide diagnostic information, it is a rapid, minimally painful and safe procedure which can be repeated at regular, short intervals. This would be highly beneficial for longitudinal monitoring during HBV therapy, as has been undertaken in HCV.^16^

However, efforts should be made to standardise FNA, including both the practical procedure at the patient bedside and the processing of the sample. Improving and optimising sampling techniques may allow researchers to gain more information with different techniques (*e.g.* digital droplet PCR for the quantification of cccDNA and viral RNA, flow cytometry and cytometry by time of flight for the phenotypic and functional analysis of immune cells, RNA-sequencing for an unsupervised analysis of transcriptomics, chromatin immunoprecipitation-sequencing for analysis of the viral reservoir). This would ultimately lead to viral and immune assay improvement, unifying clinical trial data for the HBV cure programme. In line with this, an eagerly awaited platform for such harmonisation is in the development process.

### Are immunological and virological markers evenly distributed in the liver?

Noting the importance of sampling the liver, it is still key to remember that with liver biopsy or FNA only a very small proportion of the liver is sampled. Thus, interpretations of how this impacts overall viral and immune-related pathology in the HBV-infected liver needs to be considered. In addition, liver immune cells can be rapidly and dramatically changed during inflammation, which can impact disease outcomes. In cirrhotic livers, the transcriptional activity of viral markers may be different to non-cirrhotic livers. Liver lobule zonation, including oxygen exposure and metabolic zonation, may be altered during chronic liver disease,^17^ which may impact interactions between infected hepatocytes and immune cells. Indeed, it has been shown that HBV antigen presentation can be heterogeneous; consequently, T cells of different HBV specificities may have different antiviral efficacies.^18^ This patchy distribution of immune cells along with the transcriptional activity of viral markers^19^ will likely impact our understanding of virological and immunological mechanisms in HBV, as well as affecting clinical outcomes. These details require further consideration when aiming to develop novel immune and viral biomarkers for HBV cure.

## Dynamics of cccDNA replenishment and maintenance

### What is the half-life of the HBV cccDNA in the human liver?

The understanding of the half-life of functional cccDNA – *i.e.* transcriptionally active and capable of supporting viral replication and/or production of viral proteins – is critical because the duration needed to eliminate functional cccDNA, or to reduce it to low enough levels that immune or other mechanisms render it clinically irrelevant, dictates the duration of antiviral therapy. In turn, this defines the types of therapies that could achieve a functional cure, with longer half-lives favouring treatments that induce degradation and/or permanent silencing of cccDNA, and shorter half-lives permitting use of treatments that block cccDNA production (*i.e*. totally blocking HBV replication for 24 weeks would reduce cccDNA levels by >16 million-fold if the cccDNA half-life were 1 week, whereas levels would be reduced by only 16-fold if the half-life were 6 months). While the exact determination of the lifespan of cccDNA remains challenging, a recent estimate of its half-life of 6.9–21.8 weeks^20^ is highly informative regarding the likely duration of therapy needed to achieve a functional cure.

Gaining insights on the degradation rate of functional cccDNA will also be important to guide the design of curative therapy trials and to determine when it is safe to stop therapy. As discussed above, the assessment of the intrahepatic viral reservoir would provide such information. Indeed, the treatment course must be anticipated to perform statistical power analyses, estimate patient recruitment/retention strategies, rates and costs, and to define endpoints.

Studies with chimeric mice carrying humanised livers will continue to be essential even as we move into clinical trials, as animal models permit the highly invasive studies required to define the mechanisms leading to cccDNA destruction or silencing, and hence how to achieve functional cure. Human studies confirming the effects of experimental therapies on cccDNA will be needed because chimeric mice do not fully recapitulate human biology and lack adaptive immunity. Maximising information obtainable from patients through core liver biopsies and non- or less-invasive sampling methods (such as FNA and non-invasive viral biomarkers), particularly studies to measure cccDNA in longitudinal studies, will be essential to define curative therapies and develop practical endpoints for treatment regimens.

### How does the half-life of cccDNA change during antiviral therapy?

The cccDNA turnover rate is very likely to change from baseline during antiviral therapy because production rates of HBV DNA, cccDNA, and antigens will be altered and such alterations may affect the intrinsic degradation rate of cccDNA and/or alter immune responses to infected hepatocytes. The rate of cccDNA decline will be faster if either the cccDNA production rate is reduced and/or if its degradation/silencing rate is increased. Altering either of these values would change the apparent cccDNA half-life during therapy.

Different candidate curative drug combinations are likely to have varying effects on the half-life of cccDNA due to their different mechanisms of action and impact on infected hepatocyte survival. For example, replication inhibitors are likely to primarily affect the cccDNA production rate, whereas immune-stimulating agents may stimulate cccDNA silencing and/or degradation, or eliminate infected hepatocytes.

Studies to measure the apparent half-life of functional cccDNA during therapy will be very informative in defining clinical endpoints, particularly safe stopping rules. Such studies will require longitudinal monitoring of functional cccDNA. Optimally, such studies would use less-invasive sampling techniques to permit cccDNA monitoring during clinical trials; thus, efforts to develop and validate the performance of techniques that are less invasive than a core liver biopsy, such as FNAs, will be of high priority. cccDNA monitoring may also require the improvement of current methods to monitor HBV using single-cell technologies^21^^,^^22^ for HBV genome tracking and sequencing, as well as transcript analysis and viral antigen expression if intracellular levels of HBV products are highly variable between cells and/or at the limit of detection for current assays.

### What is the contribution of *de novo* infection of naive hepatocytes in cccDNA maintenance in the human liver?

The paradigm for HBV cccDNA maintenance in hepatocytes has been that cccDNA can be made from either the viral DNA delivered into a cell by an infecting virion (*de novo* formation) or through an intracellular route in which the newly synthesised genomes made by reverse transcription are trafficked to the nucleus without being secreted (intracellular amplification or “recycling”). However, *in vivo* data using the highly effective HBV entry inhibitor bulevirtide (Hepcludex) in HBV-infected chimeric mice imply that cccDNA formation is largely, or perhaps exclusively, made via the *de novo* route.^23^ An entry inhibitor would do this by blocking *de novo* cccDNA synthesis to a degree that could not be compensated for by intracellular amplification. Human studies to define the role of *de novo* infection on cccDNA maintenance will be important because they will reveal if drugs blocking viral entry will be needed in future curative combination therapies. Thus, tracking the number of infected cells and cccDNA reservoir during these therapies will be of critical importance.

## Impact of integrated HBV DNA on novel cure strategies

### Is HBsAg loss the best endpoint for new therapies?

HBV DNA integrates in the early stages of infection, *i.e.* in patients with acute hepatitis and in the early “immune tolerant” phase of chronic infection.^24^^,^^25^ This process continues throughout all the phases of a chronic infection. Current evidence indicates that circulating HBsAg may be produced from both cccDNA and integrated HBV DNA and that, in HBeAg-negative patients, integrated HBV DNA may represent the most important source of HBsAg.[26], [27], [28], [29] This implies that the efficacy of new therapeutic approaches, in particular those targeting cccDNA, may not be mirrored by a reduction/loss of HBsAg levels. This is also related to the fact that no diagnostic assay is currently able to distinguish between HBsAg derived from integrated HBV and that derived from cccDNA. Therefore, basic studies to identify possible differences in the composition and source (integrated DNA *vs*. cccDNA) of surface proteins included in viral and subviral particles would be required. In view of these considerations, the use of HBsAg as a surrogate marker for HBV functional cure should be re-evaluated. Thus, the assessment of the intrahepatic cccDNA reservoir, in terms of total amount, its epigenetic status and the number of infected cells is critical. This has been demonstrated, at least in proof-of-concept studies of new therapeutic strategies.[29], [30], [31] Non-invasive biomarkers evaluating the size of the transcriptionally active cccDNA pool require development and validation for large-scale clinical trials.

### How can NAs affect viral integrants?

Important data have recently been provided showing that suppression of viral replication in patients with CHB undergoing long-term NA treatment is significantly associated with a reduced extent of HBV DNA integration,^32^ a decreased number of distinct expressed integrations^33^ and a reduction in hepatocyte clone size.^32^ Interestingly, reduction in transcriptionally active HBV integrations after 3 years of treatment with tenofovir disoproxil fumarate was associated with a decrease in dysregulated genes, including those implicated in hepatocellular carcinogenesis.^33^ The exact mechanisms through which NAs may affect viral integrants remain elusive. There is *in vitro* evidence demonstrating that tenofovir disoproxil fumarate cannot block *de novo* HBV DNA integration in infected hepatocytes.^34^ It also appears improbable that NAs would directly affect existing HBV integrants. The reported reduction in viral integration, in all likelihood, can be attributed to the strong and sustained suppression of viral replication, and production of double-stranded linear HBV DNA (the template of integration), by long-term treatment with NAs. Possibly, the abatement of viremia levels by NAs strongly limits *de novo* infection with the consequent decline over time of the number of hepatocytes harbouring viral integrations. Therefore, replacement of infected hepatocytes with regenerated uninfected cells may account for the significant reduction in viral integrations observed in NA-treated patients. Overall, these results support data from previous studies,^10^^,^^35^^,^^36^ which highlighted the need to reconsider the recommended criteria to initiate antiviral treatment for CHB, suggesting that efficient antiviral therapy should be considered early in adult patients with a high level of HBV viremia even in the absence of severe liver damage in order to limit the number of HBV integrations, minimise genetic damage, and fight against direct HBV-related carcinogenesis. Thus, the question of whether the burden of viral genome integration could be assessed longitudinally on FNA samples will need to be addressed to inform treatment guidelines on the currently available antivirals.

### Should novel HBV cure strategies target integrated HBV DNA in addition to cccDNA?

HBV DNA integration may favour persistence of viral infection and drive hepatocellular carcinogenesis.^25^^,^^37^ As a stable intrahepatic source of immunomodulatory HBsAg and C-terminally truncated HBx,^11^^,^^24^ HBV integration might favour both viral persistence and immune tolerance.^11^ In particular, the preservation of HBsAg production independently of HBV replication suggest that HBV integrants may sustain the suppression of the antiviral immune response. This assumption is supported by evidence demonstrating that durable presentation of high levels of HBsAg induces T-cell exhaustion.^3^^,^^38^^,^^39^ Together, functional and deletional tolerance may synergise in preventing the clearance of HBV infection. Thus, HBV integration may contribute to both sustaining the suppression of the HBV-specific immune response and supporting the persistence of viral infection.

It has been estimated that for any given gene, there are about 500 hepatocytes in the liver containing an HBV DNA integration.^34^ Therefore, every patient exposed to HBV, including those that do not meet treatment guidelines criteria could be at an increased risk of HCC.^37^ Although most HBV integrations are passenger events and do not have any functional consequences, some of them can behave as driver events favouring HCC initiation both in cirrhotic or non-cirrhotic livers. Indeed, recent studies have provided evidence indicating that HBV DNA integration may alter the transcriptome profile of human hepatocytes and activate HCC-related pathways in patients with CHB and limited liver disease progression, low-to-moderate viremia levels, and minimally raised serum alanine aminotransferase concentrations.^33^^,^^40^ These studies demonstrate that sustained inhibition of HBV replication by readily available novel cure strategies and by early therapeutic intervention with currently available antiviral agents can decrease the extent of viral integrations, reduce genetic damage to hepatocytes, and minimise the promotion of carcinogenesis. Furthermore, evaluation of the pattern of viral integration should be considered when developing new therapeutic strategies, in particular those targeting viral RNA or those that directly target cccDNA. Analysing DNA and RNA sequence data from liver samples obtained from core biopsies or from longitudinally collected FNA samples will give important insights on this question. In-depth comparisons of circulating viral RNA sequences with hepatic DNA and RNA sequences would also help to determine if these “liquid biopsies” could represent a surrogate for the evaluation of the viral integration burden and its correlation with HCC risk.^41^ All this indicates that the evaluation of HBV integration may have a significant impact on future cure strategies, with the following underlying questions remaining: i) the impact of HBV integration sequences on the choice and design of approaches targeting viral transcripts, ii) the impact of novel strategies on the viral integration burden and subsequent HCC risk reduction and iii) the best technological approaches to monitor viral genome integration.

## Transcriptional activity of cccDNA

### How is transcription of cccDNA regulated during the different phases of HBV infection?

Transcription from cccDNA is tightly regulated and is subject to epigenetic regulation.^42^ However, most studies on cccDNA transcription originate from cell culture experiments and only focus on specific aspects of transcriptional regulation. A comprehensive understanding of cccDNA transcription, in particular in patients, is still lacking. To design epigenetic therapy approaches, we must understand the transcriptional processes that take place in patients over the course of the disease. In particular, the mechanism(s) leading to silencing of cccDNA transcription, which could potentially take place after clearance of acute infection or at late stages of HBeAg-negative hepatitis, need to be understood to design therapeutic strategies with the aim of silencing cccDNA. Furthermore, it is likely that not all cccDNA molecules have the same epigenetic status within the liver at a given time of infection and this may evolve in a dynamic manner. The co-existence of transcriptionally active and epigenetically silenced cccDNA will need to be investigated as well as its evolution in response to the liver microenvironment.

Future studies should analyse the epigenetic landscape, the role of associated transcriptional regulators as well as the nuclear localisation and the interaction with viral and host factors, such as the interaction of HBx and the SMC5/6 complex. Commonly used methods for the analysis of transcriptional regulation include chromatin immunoprecipitation followed by qPCR or sequencing and chromosome conformation capture technologies. However, applying these techniques to HBV research is complicated by the low abundance of cccDNA molecules and the high abundance of sequence similar HBV DNA intermediates and HBV DNA integrations in the host genome that might be co-detected in these assays. Since these assays require frozen liver core biopsies, many aspects will also have to be addressed in preclinical animal models harbouring transcriptionally active cccDNA, such as human liver chimeric mice or mice equipped with recombinant cccDNA.^43^ These assays will have to be miniaturised to be applicable to FNA samples.

Non-invasive serum biomarkers will be helpful to evaluate cccDNA activity by judging its transcriptional output, the viral RNAs. pgRNA and precore mRNA are over-length viral RNAs that originate from cccDNA but not HBV integrations and can thus be used to monitor cccDNA transcription, together with their protein products (HBeAg, HBcrAg).^44^

### How do antiviral treatments affect transcriptional regulation of the cccDNA?

It is important to investigate if and how antivirals affect cccDNA transcription – both approved and novel antivirals – in order to harness their potential for HBV cure therapies and determine the best combination treatments. Unfortunately, very limited data from patient liver biopsies are available^22^^,^^45^^,^^46^ and more information from epigenetic studies in liver biopsies or FNA samples is urgently needed. On-treatment sampling of the liver by FNA will be helpful to analyse pgRNA/precore mRNA as a surrogate for the transcriptional activity of cccDNA. Serum biomarkers can inform us regarding the transcriptional activity of cccDNA, but it must be taken into account that changes at other steps of the viral life cycle will also be reflected in the level of serum biomarkers. Hence, preclinical models to elucidate the mode of action of the investigated drug will be similarly important.

### What is the mechanism of HBV reactivation after treatment cessation or immunosuppression?

To answer the question of whether treatment strategies should primarily focus on cccDNA silencing or on its eradication, it will be important to gain a better understanding of the mechanisms of transcriptional silencing and reactivation. While there is clinical evidence that transcriptionally silent cccDNA molecules exist^21^ with an increase of transcriptional silencing during the transition to HBeAg-negative chronic hepatitis,^28^^,^^47^ it is not clear if silencing is absolute and how these silenced molecules could become reactivated. On the other hand, there is evidence that low levels of cytotoxic T-cell responses are maintained for decades in patients who have recovered from acute infection.^48^ One possible explanation for this observation would be that traces of active virus persist in the liver, which are able to continuously or intermittently activate cytotoxic T-cell responses. The observation that the loss of B-cell responses after rituximab-based chemotherapy is associated with high rates of HBV relapse also highlights the role of antibodies in the control of persistent infection.^49^ Therefore, it is likely that the reactivation of HBV infection following treatment cessation or immunosuppression is caused by ramping up of very low ongoing *de novo* infections (from active cccDNA molecules) after immune or drug pressure rather than by reactivation of silenced cccDNA. This persistent replication model would also explain how cccDNA could be maintained for decades beyond the lifespan of hepatocytes. Note that both models for HBV persistence (persistent replication or silenced cccDNA) are not mutually exclusive as the epigenetic status of a single cccDNA molecule may evolve in a dynamic manner in response to the cellular and liver microenvironment, leading to intermittent low-level replication.

Addressing this question in patients will be challenging because of the need for liver biopsies in functionally cured and reactivated patients; thus, FNA, serum biomarkers and preclinical animal models will be important surrogate samples.

## Relevance of intrahepatic and blood-derived immunity on cure strategies

### Which immune parameters can be assessed in the circulation and which require liver sampling?

The major advantage of blood sampling is the accessibility and possibility to easily quantify the circulating levels of immune cells or immunologically active molecules. This is exemplified by the measurement of anti-HBs antibody titres to assess vaccine-induced immunity. Many studies previously relied on blood sampling, which provided important insights into HBV pathogenesis.^50^ Recent data, however, have highlighted that the composition of cellular immune infiltrates is different between the liver and circulation.^50^ In addition, the hepatic immune cell infiltrate may depend on the local HBV transcriptional activity and the fibrotic environment. Given that HBV transcripts and fibrotic areas are not evenly distributed among the liver parenchyma (see above), it is highly likely that the intrahepatic immune infiltrate has a similarly patchy distribution. Thus, efforts to sample blood and liver tissue simultaneously to establish circulating correlates of intrahepatic immunity may suffer from the heterogeneous distribution of immune cells within the liver. However, it has become evident that several immune cell populations, such as tissue-resident T cells, are hardly found in the circulation and will require liver sampling to understand their role in controlling HBV infection.^15^ Detailed analyses of HBV-specific T-cell exhaustion have also highlighted the importance of studying intrahepatic immune cells because functional studies of T cells from the blood compartment gave only partial information.^15^ It is clear that both blood and liver sampling have limitations with regards to gaining an overall picture of HBV immunity, which should be considered when designing immunological studies. Indeed, for immune monitoring purposes, or identification of T-cell epitopes and/or viral immune escape mutations, liver sampling may not be regularly required. In contrast, assessment of the re-education of the liver immune compartment in proof-of-concept clinical trials and the identification of immunological therapeutic targets might be more relevant at the site of infection.

### What are the key parameters that should be studied when liver samples are available?

The development of novel technologies to assess the transcriptional activity, epigenetic regulation, metabolic fitness and spatial distribution of immune cells at the single-cell level is providing great opportunities to dissect antiviral immune responses in detail.[51], [52], [53] This might prove particularly helpful in the context of HBV infection, where we are dealing with small immune cell populations in small tissue samples (be it FNA or core biopsy). These novel technologies will improve the identification of therapeutic targets, as has been discussed. However, it remains difficult at times to translate these “deep phenotyping data” into functional outputs. In this context, mouse models will remain instrumental as they can help to close the gap between correlative analyses and proof of causality, providing important mechanistic evidence which may aid future drug design.

The key parameters to be studied in liver samples certainly depend on tissue quantity, the underlying immunological question and the context in which the tissue has been obtained (*e.g*., clinical study with a specific focus). It is therefore important to define and specify the questions that are being asked when liver sampling is performed. This appears particularly important in the case of FNA, where the limited volume of material available for analysis must be taken into account. Viral parameters, such as cccDNA or pgRNA, that act as surrogates of transcriptional activity within hepatocytes can be measured from the same FNA sample that immunological analyses are performed on; the extent of such virologic and immunological analyses may require several passes for liver cell aspiration to obtain sufficient material.

In contrast to peripheral blood samples, where one could make a convincing argument that only HBV-specific immune parameters should be analysed; assessment of intrahepatic immunity should, in addition to HBV-specific immune parameters, also include non-virus-specific immune cells that may be pertinent to immune pathogenesis in a bystander manner. These include natural killer, natural killer T and unconventional T cells (mucosal-associated invariant T, gamma-delta) as these are also abundant at the site of infection. At this stage, any novel insights into intrahepatic immunity in well described cohorts is of great importance and would broaden our understanding of intrahepatic immunity to HBV during CHB.

### Which immune-mediated functions most efficiently interfere with transcriptional activity and cccDNA stability?

This is a key question that is part of the ‘holy grail’ of information needed to design cures for CHB. While it is well known that cytokines such as interferons can interfere with the HBV machinery,^54^ several questions surrounding these observations remain unanswered, such as: what are the required concentrations? which co-factors need to be involved? what are the cellular sources of the individual cytokines? how much cytotoxicity is required to control HBV infection and eventually lead to functional cure without causing overt hepatic immunopathology? The latter also includes the question of whether we can differentiate between good (antiviral) and bad (immunopathology) immune cell functions? In addition, the precise immunological mechanisms that are required to clear HBV infection might depend on the number of infected hepatocytes, *i.e.* eliminating residually infected cells *vs.* elimination of large numbers of HBV-infected cells, where the question regarding cytotoxicity is particularly important.

## Perspectives: Analysis of the liver compartment to assist drug development

Studying the intrahepatic viral reservoir and the resulting immune responses in clinical studies will be central to better understand the mechanism of viral persistence and assess the antiviral and immune activity of novel treatment strategies in development. This highlights the importance of identifying the optimal clinical procedure to obtain liver samples, and of validating FNA as a less-invasive sampling method compared to conventional core liver biopsy. Improved molecular virology and immunology techniques will be required to enable sensitive assessment at the single-cell level and all these techniques will need to be validated prior to their application in clinical research and drug development.

This should provide opportunities to integrate intrahepatic virological and immunological data, which has not been done so far in clinical studies. Assessing the re-education of the liver immune microenvironment and the cccDNA reservoir should provide extremely useful information to guide the development of novel direct-acting antivirals and immune modulators, as well their use in treatment combinations. Analysing the liver compartment in sub studies of larger clinical trials or in proof-of-concept trials will be particularly important when new modes of action or new combinations are being investigated. In this respect, FNA currently represents a promising research tool that should facilitate sequential/longitudinal assessment in these clinical studies. It should also provide very important information for the validation of novel viral and immunological biomarkers that may assist large-scale clinical trials. With the improvement of molecular and cellular biology technologies and the concomitant advances in drug discovery/development, it will be exciting to see how these liver studies will inform the HBV cure programme.

## Financial support

TB is supported by the 10.13039/501100001659Deutsche Forschungsgemeinschaft (DFG, German Research Foundation) - project 272983813 - TP04. USG is supported by grant funding from an Academy of Medical Sciences Starter Grant (SGL021/1030), Seedcorn funding Rosetrees/Stoneygate Trust (A2903) and Early Career Research Award from The Medical Research Foundation (MRF-044-0004-F-GILL-C0823). FZ is supported by the ANRS-MIE HBV cure task force.

## Authors' contributions

Manuscript concept: TB, FZ; drafting of the manuscript: TB, USG, LA, TP, JET, FZ

## Conflict of interest

The authors declare no conflicts of interest that pertain to this work.

Please refer to the accompanying ICMJE disclosure forms for further details.
