# Supplementary material for: Assessing immunological and virological responses in the liver: Implications for the cure of chronic hepatitis B virus infection
Source: JHEP Rep. 2022 Apr 2;4(6):100480. doi: 10.1016/j.jhepr.2022.100480 (PMC9039841; doi:10.1016/j.jhepr.2022.100480)
Supplement: Multimedia component 1 [file mmc1.pdf]

ICMJE DISCLOSURE FORM

Date: 3/17/2022

Your Name: Lena Allweiss

Manuscript Title: Assessing immunological and virological responses in the liver: implications for the cure of chronic hepatitis B virus infection

Manuscript Number (if known): JHEPR-D-21-00370

In the interest of transparency, we ask you to disclose all relationships/activities/interests listed below that are related to the content of your manuscript. "Related" means any relation with for-profit or not-for-profit third parties whose interests may be affected by the content of the manuscript. Disclosure represents a commitment to transparency and does not necessarily indicate a bias. If you are in doubt about whether to list a relationship/activity/interest, it is preferable that you do so.

The author's relationships/activities/interests should be defined broadly. For example, if your manuscript pertains to the epidemiology of hypertension, you should declare all relationships with manufacturers of antihypertensive medication, even if that medication is not mentioned in the manuscript.

In item #1 below, report all support for the work reported in this manuscript without time limit. For all other items, the time frame for disclosure is the past 36 months.

		Name all entities with whom you have this relationship or indicate none (add rows as needed)	Specifications/Comments (e.g., if payments were made to you or to your institution)
Time frame: Since the initial planning of the work			
1	All support for the present manuscript (e.g., funding, provision of study materials, medical writing, article processing charges, etc.) **No time limit for this item.**	☒ None	
			Click the tab key to add additional rows.
Time frame: past 36 months			
2	Grants or contracts from any entity (if not indicated in item #1 above).	☒ **None**	
3	Royalties or licenses	☒ **None**	

		Name all entities with whom you have this relationship or indicate none (add rows as needed)	Specifications/Comments (e.g., if payments were made to you or to your institution)
**4**	Consulting fees	☒ **None**	
**5**	Payment or honoraria for lectures, presentations, speakers bureaus, manuscript writing or educational events	☒ **None**	
**6**	Payment for expert testimony	☒ **None**	
**7**	Support for attending meetings and/or travel	☒ **None**	
**8**	Patents planned, issued or pending	☒ **None**	
**9**	Participation on a Data Safety Monitoring Board or Advisory Board	☒ **None**	
**10**	Leadership or fiduciary role in other board, society, committee or advocacy group, paid or unpaid	☒ **None**	

		Name all entities with whom you have this relationship or indicate none (add rows as needed)	Specifications/Comments (e.g., if payments were made to you or to your institution)
**11**	Stock or stock options	☒ **None**	
**12**	Receipt of equipment, materials, drugs, medical writing, gifts or other services	☒ **None**	
**13**	Other financial or non-financial interests	☒ **None**	

Please place an "X" next to the following statement to indicate your agreement:

☒ I certify that I have answered every question and have not altered the wording of any of the questions on this form.

ICMJE DISCLOSURE FORM

Date: 3/8/2022

Your Name: Tobias Boettler

Manuscript Title: Assessing immunological and virological responses in the liver: implications for the cure of chronic hepatitis B virus infection

Manuscript Number (if known): JHEPR-D-21-00370

In the interest of transparency, we ask you to disclose all relationships/activities/interests listed below that are related to the content of your manuscript. "Related" means any relation with for-profit or not-for-profit third parties whose interests may be affected by the content of the manuscript. Disclosure represents a commitment to transparency and does not necessarily indicate a bias. If you are in doubt about whether to list a relationship/activity/interest, it is preferable that you do so.

The author's relationships/activities/interests should be defined broadly. For example, if your manuscript pertains to the epidemiology of hypertension, you should declare all relationships with manufacturers of antihypertensive medication, even if that medication is not mentioned in the manuscript.

In item #1 below, report all support for the work reported in this manuscript without time limit. For all other items, the time frame for disclosure is the past 36 months.

		Name all entities with whom you have this relationship or indicate none (add rows as needed)	Specifications/Comments (e.g., if payments were made to you or to your institution)
**Time frame: Since the initial planning of the work**			
1	All support for the present manuscript (e.g., funding, provision of study materials, medical writing, article processing charges, etc.) **No time limit for this item.**	☐ None	
		Deutsche Forschungsgemeinschaft	Research support, payment to institution
			Click the tab key to add additional rows.
**Time frame: past 36 months**			
**2**	Grants or contracts from any entity (if not indicated in item #1 above).	☒ **None**	
**3**	Royalties or licenses	☒ **None**	

		Name all entities with whom you have this relationship or indicate none (add rows as needed)	Specifications/Comments (e.g., if payments were made to you or to your institution)
**4**	Consulting fees	☐ **None**	
		Gilead Sciences	
**5**	Payment or honoraria for lectures, presentations, speakers bureaus, manuscript writing or educational events	☒ **None**	
**6**	Payment for expert testimony	☒ **None**	
**7**	Support for attending meetings and/or travel	☒ **None**	
**8**	Patents planned, issued or pending	☒ **None**	
**9**	Participation on a Data Safety Monitoring Board or Advisory Board	☐ **None**	
		Gilead Sciences	
**10**	Leadership or fiduciary role in other board, society, committee or advocacy group, paid or unpaid	☐ **None**	
		European Association for the study of the liver	Scientific committee/Governing Board member

		Name all entities with whom you have this relationship or indicate none (add rows as needed)	Specifications/Comments (e.g., if payments were made to you or to your institution)
**11**	Stock or stock options	☒ **None**	
**12**	Receipt of equipment, materials, drugs, medical writing, gifts or other services	☒ **None**	
**13**	Other financial or non-financial interests	☒ **None**	

Please place an "X" next to the following statement to indicate your agreement:

☒ I certify that I have answered every question and have not altered the wording of any of the questions on this form.

ICMJE DISCLOSURE FORM

Date:__16th March 2022 ____
Your Name:_Dr Upkar S. Gill ____
Manuscript Title:_ Assessing immunological and virological responses in the liver: implications for the cure of chronic hepatitis B virus infection ____
Manuscript number (if known):___ JHEPR-D-21-00370R1 ______________________________________

In the interest of transparency, we ask you to disclose all relationships/activities/interests listed below that are related to the content of your manuscript. "Related" means any relation with for-profit or not-for-profit third parties whose interests may be affected by the content of the manuscript. Disclosure represents a commitment to transparency and does not necessarily indicate a bias. If you are in doubt about whether to list a relationship/activity/interest, it is preferable that you do so.

The following questions apply to the author's relationships/activities/interests as they relate to the current manuscript only.

The author's relationships/activities/interests should be defined broadly. For example, if your manuscript pertains to the epidemiology of hypertension, you should declare all relationships with manufacturers of antihypertensive medication, even if that medication is not mentioned in the manuscript.

In item #1 below, report all support for the work reported in this manuscript without time limit. For all other items, the time frame for disclosure is the past 36 months.

		**Name all entities with whom you have this relationship or indicate none (add rows as needed)**	**Specifications/Comments (e.g., if payments were made to you or to your institution)**
		**Time frame: Since the initial planning of the work**	
1	All support for the present manuscript (e.g., funding, provision of study materials, medical writing, article processing charges, etc.) **No time limit for this item.**	____None	
		**Time frame: past 36 months**	
2	Grants or contracts from any entity (if not indicated in item #1 above).	____None	
3	Royalties or licenses	____None	
4	Consulting fees	____None	

5	Payment or honoraria for lectures, presentations, speakers bureaus, manuscript writing or educational events	____None	
6	Payment for expert testimony	____None	
7	Support for attending meetings and/or travel	____None	
8	Patents planned, issued or pending	____None	
9	Participation on a Data Safety Monitoring Board or Advisory Board	____None	
10	Leadership or fiduciary role in other board, society, committee or advocacy group, paid or unpaid	____None	
11	Stock or stock options	____None	
12	Receipt of equipment, materials, drugs, medical writing, gifts or other services	____None	
13	Other financial or non-financial interests	____None	

Please place an "X" next to the following statement to indicate your agreement:

__X_ I certify that I have answered every question and have not altered the wording of any of the questions on this form.

ICMJE DISCLOSURE FORM

Date:___March 14, 2022____
Your Name:________ John Tavis, PhD ____
Manuscript Title:___ Assessing immunological and virological responses in the liver: implications for the cure of chronic hepatitis B virus infection ___
Manuscript number (if known):___ JHEPR-D-21-00370R1 ____________________________________

In the interest of transparency, we ask you to disclose all relationships/activities/interests listed below that are related to the content of your manuscript. "Related" means any relation with for-profit or not-for-profit third parties whose interests may be affected by the content of the manuscript. Disclosure represents a commitment to transparency and does not necessarily indicate a bias. If you are in doubt about whether to list a relationship/activity/interest, it is preferable that you do so.

The following questions apply to the author's relationships/activities/interests as they relate to the current manuscript only.

The author's relationships/activities/interests should be defined broadly. For example, if your manuscript pertains to the epidemiology of hypertension, you should declare all relationships with manufacturers of antihypertensive medication, even if that medication is not mentioned in the manuscript.

In item #1 below, report all support for the work reported in this manuscript without time limit. For all other items, the time frame for disclosure is the past 36 months.

		**Name all entities with whom you have this relationship or indicate none (add rows as needed)**	**Specifications/Comments (e.g., if payments were made to you or to your institution)**
		**Time frame: Since the initial planning of the work**	
1	All support for the present manuscript (e.g., funding, provision of study materials, medical writing, article processing charges, etc.) **No time limit for this item.**	____None	
		**Time frame: past 36 months**	
2	Grants or contracts from any entity (if not indicated in item #1 above).	____None	
3	Royalties or licenses	____None	
4	Consulting fees	Antios Therapeutics	Scientific Advisor; payment to me

5	Payment or honoraria for lectures, presentations, speakers bureaus, manuscript writing or educational events	____None	
6	Payment for expert testimony	____None	
7	Support for attending meetings and/or travel	____None	
8	Patents planned, issued or pending	____None	
9	Participation on a Data Safety Monitoring Board or Advisory Board	____None	
10	Leadership or fiduciary role in other board, society, committee or advocacy group, paid or unpaid	_Hepatitis B Foundation	Scientific Advisor; unpaid
		HBV Forum	Governing board member; unpaid
		International HBV meeting	Chairman, Scientific Advisory Council; unpaid
		ICE-HBV	Incoming Chairman, Governing Board; unpaid
11	Stock or stock options	____None	
12	Receipt of equipment, materials, drugs, medical writing, gifts or other services	____None	
13	Other financial or non-financial interests	____None	

Please place an "X" next to the following statement to indicate your agreement:

___ I certify that I have answered every question and have not altered the wording of any of the questions on this form.

ICMJE DISCLOSURE FORM

Date: 14/03/2022
Your Name: Teresa Pollicino
Manuscript Title: Assessing immunological and virological responses in the liver: implications for the cure of chronic hepatitis B virus infection
Manuscript number (if known): JHEPR-D-21-00370R1

In the interest of transparency, we ask you to disclose all relationships/activities/interests listed below that are related to the content of your manuscript. "Related" means any relation with for-profit or not-for-profit third parties whose interests may be affected by the content of the manuscript. Disclosure represents a commitment to transparency and does not necessarily indicate a bias. If you are in doubt about whether to list a relationship/activity/interest, it is preferable that you do so.

The following questions apply to the author's relationships/activities/interests as they relate to the current manuscript only.

The author's relationships/activities/interests should be defined broadly. For example, if your manuscript pertains to the epidemiology of hypertension, you should declare all relationships with manufacturers of antihypertensive medication, even if that medication is not mentioned in the manuscript.

In item #1 below, report all support for the work reported in this manuscript without time limit. For all other items, the time frame for disclosure is the past 36 months.

		Name all entities with whom you have this relationship or indicate none (add rows as needed)	Specifications/Comments (e.g., if payments were made to you or to your institution)
**Time frame: Since the initial planning of the work**			
1	All support for the present manuscript (e.g., funding, provision of study materials, medical writing, article processing charges, etc.) **No time limit for this item.**	X None	
**Time frame: past 36 months**			
2	Grants or contracts from any entity (if not indicated in item #1 above).	X None	
3	Royalties or licenses	X None	
4	Consulting fees	X None	

5	Payment or honoraria for lectures, presentations, speakers bureaus, manuscript writing or educational events	____None<br>Honoraria for lectures from Gilead Sciences
6	Payment for expert testimony	__X__None
7	Support for attending meetings and/or travel	__X__None
8	Patents planned, issued or pending	__X__None
9	Participation on a Data Safety Monitoring Board or Advisory Board	__X__None
10	Leadership or fiduciary role in other board, society, committee or advocacy group, paid or unpaid	__X__None
11	Stock or stock options	__X__None
12	Receipt of equipment, materials, drugs, medical writing, gifts or other services	__X__None
13	Other financial or non-financial interests	__X__None

Please place an "X" next to the following statement to indicate your agreement:

_X_ **I certify that I have answered every question and have not altered the wording of any of the questions on this form.**

ICMJE DISCLOSURE FORM

Date:___March 16, 2022____
Your Name:___Fabien Zoulim____
Manuscript Title:__ Assessing immunological and virological responses in the liver: implications for the cure of chronic hepatitis B virus infection
____
Manuscript number (if known):______ JHEPR JHEPR-D-21-00370R1

In the interest of transparency, we ask you to disclose all relationships/activities/interests listed below that are related to the content of your manuscript. "Related" means any relation with for-profit or not-for-profit third parties whose interests may be affected by the content of the manuscript. Disclosure represents a commitment to transparency and does not necessarily indicate a bias. If you are in doubt about whether to list a relationship/activity/interest, it is preferable that you do so.

The following questions apply to the author's relationships/activities/interests as they relate to the current manuscript only.

The author's relationships/activities/interests should be defined broadly. For example, if your manuscript pertains to the epidemiology of hypertension, you should declare all relationships with manufacturers of antihypertensive medication, even if that medication is not mentioned in the manuscript.

In item #1 below, report all support for the work reported in this manuscript without time limit. For all other items, the time frame for disclosure is the past 36 months.

		**Name all entities with whom you have this relationship or indicate none (add rows as needed)**	**Specifications/Comments (e.g., if payments were made to you or to your institution)**
**Time frame: Since the initial planning of the work**			
1	All support for the present manuscript (e.g., funding, provision of study materials, medical writing, article processing charges, etc.) **No time limit for this item.**	____None	
**Time frame: past 36 months**			
2	Grants or contracts from any entity (if not indicated in item #1 above).	Beam	To INSERM
		JnJ	To INSERM
		Assembly	To INSERM
3	Royalties or licenses	____None	

4	Consulting fees	Aligos, Antios, Arbutus, Assembly, Enanta, Enochian, GSK, VIR, Viravaxx	Personal
5	Payment or honoraria for lectures, presentations, speakers bureaus, manuscript writing or educational events	____Gilead	Personal
6	Payment for expert testimony	____None	
7	Support for attending meetings and/or travel	____None	
8	Patents planned, issued or pending	____None	
9	Participation on a Data Safety Monitoring Board or Advisory Board	____None	
10	Leadership or fiduciary role in other board, society, committee or advocacy group, paid or unpaid	____None	
11	Stock or stock options	____None	
12	Receipt of equipment, materials, drugs, medical writing, gifts or other services	____None	
13	Other financial or non-financial interests	____None	

Please place an "X" next to the following statement to indicate your agreement:

x_ I certify that I have answered every question and have not altered the wording of any of the questions on this form.
